# The Effect of Decreased Ca^++^/Mg^++^ ATPase Activity on *Lactobacillus delbrueckii* subsp. *bulgaricus* sp1.1 Survival during Spray Drying

**DOI:** 10.3390/foods12040787

**Published:** 2023-02-13

**Authors:** Jialei Sun, Wenjing Cai, Yu Wang, Haiyue Niu, Xi Chen, Xue Han

**Affiliations:** School of Chemistry and Chemical Engineering, Harbin Institute of Technology, Harbin 150000, China

**Keywords:** *Lactobacillus bulgaricus*, proteome, spray drying, Ca^++^/Mg^++^ ATPase

## Abstract

Compared with the commonly used technique of freeze-drying, spray drying has lower energy costs. However, spray drying also has a fatal disadvantage: a lower survival rate. In this study, the survival of bacteria in a spray-drying tower decreased as the water content was reduced. The water content of 21.10% was the critical point for spray drying *Lactobacillus delbrueckii* subsp. *bulgaricus* (*L. bulgaricus*) sp1.1 based on sampling in the tower. Based on the relationship between the moisture content of spray drying and the survival rate, the water content of 21.10% was also the critical point for the change in the survival rate during spray drying. Proteomic analysis was used to investigate the reasons for *L. bulgaricus* sp1.1 inactivation during and after spray drying. Gene Ontology (GO) enrichment revealed that differentially expressed proteins were mainly associated with the cell membrane and transport. In particular, proteins related to metal ion transport included those involved in the transport of potassium, calcium and magnesium ions. The protein–protein interaction (PPI) network revealed that Ca^++^/Mg^++^ adenosine triphosphatase (ATPase) may be a key protein. Ca^++^/Mg^++^ ATPase activity decreased substantially during spray drying (*p* < 0.05). Supplementation with Ca^++^ and Mg^++^ significantly increased the expression of ATPase-related genes and enzyme activity (*p* < 0.05). The Ca^++^/Mg^++^ ATPase activity of *L. bulgaricus* sp1.1 was enhanced by increasing the intracellular Ca^++^ or Mg^++^ concentration, thus increasing the survival of spray-dried LAB. Bacterial survival rates were increased to 43.06% with the addition of Ca^++^ and to 42.64% with the addition of Mg^++^, respectively. Ca^++^/Mg^++^ ATPase may be the key to the damage observed in spray-dried bacteria. Furthermore, the addition of Ca^++^ or Mg^++^ also reduced bacterial injury during spray drying by enhancing the activity of Ca^++^/Mg^++^ ATPase.

## 1. Introduction

The probiotic supplement, lactic acid bacteria (LAB), is considered to contribute to the nutritional and health-promoting properties of food [1]. The commonly used technique of freeze-drying is expensive and time-consuming [2,3]. By comparison, spray drying offers the advantages of high efficiency, short drying time, a homogeneous distribution throughout the product, low cost and sustainability [4,5]. However, spray drying produces a low survival rate, mainly due to thermal inactivation and dehydration [6], which damages intracellular and membrane proteins, enzymes and ribosomes [7]. Optimization of spray drying technology to enable the production of active LAB preparations is a current research goal [8].

Most studies of bacterial inactivation have been conducted after the completion of the drying process [9]. Single-droplet drying and modeling based on single-droplet drying have been used to investigate structural changes in bacteria during spray drying [10,11]. However, there were some differences between spray drying and single-drop drying [12]. The residual activity of β-galactosidase in the single-drop experiments was different from that in a laboratory-scale spray dryer between 100 °C and 120 °C [13]. Laboratory spray drying takes 1.3–5 s [14,15], a time that is difficult to measure with hysteresis. Gong et al. established a new sampling method to study changes in the activity of *Lactobacillus* during spray drying [12]. The sampling method was used to study the changes in protein expression during spray drying. The reasons for LAB inactivation during the spray drying process were further explored.

Spray drying has been used to produce microcapsules that contain a range of bioactive compounds [16]. Microencapsulation is a unique preservation technology used as a probiotic carrier to protect against the acidic conditions of the digestive tract [17]. The carrier materials commonly used for microencapsulation include carbohydrates and proteins [18,19]. Other protective components such as lipids, divalent ions, and antioxidants were worth exploring [20,21,22]. Nevertheless, the existing microencapsulation technology still has its shortcomings. At the outset, there was no improvement in the survival rate of probiotics during storage [23,24]. In addition, the probiotics leaked out of the beads and grew in the food system. This adversely affected the sensory properties of the food products [23]. The addition of microencapsulated *Bifidobacterium longum* to yogurt had a negative impact on sensory quality due to the large particle size of the microcapsules [25]. Microcapsules were also readily dissolved in the stomach juice. The probiotics were affected by gastric acid and bile salts, causing their vitality to decrease [23,26].

Mg^++^ ions have been widely studied for their influence on bacterial homeostasis, sensing and transportation [27]. The addition of 20 mM MgCl_2_ produced a 100-fold increase in the survival rate of *Lacticaseibacillus rhamnosus* GG (LGG) under heat stress and shortened the lag phase of bacterial regrowth. Bacterial cells produce transient fluxes of Ca^++^ ions in response to environmental and physiological conditions [28], and the ions have modulatory effects on bacterial gene expression [29]. The elevated level of Ca^++^ stimulated the autophosphorylation activity of heat shock protein DnaK in *Escherichia coli* [30]. High levels of intracellular Ca^++^ may also stimulate heat shock proteins, increasing cell survival under heat stress [31]. Moreover, the addition of Ca^++^ in 10% lactose or 10% trehalose produced a 6-fold increase in survival rate after spray drying [21] without any alteration in powder morphology, IR spectrum or amorphous state. These findings indicate that the addition of Ca^++^ enhanced the intrinsic tolerance of LGG cells without altering the powder properties [21]. The mechanism by which Ca^++^ and Mg^++^ enhance bacterial intrinsic tolerance needs to be further explored.

Studies of spray drying LAB lacked an understanding of the drying process variability. At present, proteomics mainly focused on single-stress research, and there is less research on the spray drying process. Therefore, in this study, proteomics was used to study bacteria changes during spray drying and then to explore the damage mechanism of spray-dried lactic acid bacteria. Reverse transcription quantitative real-time polymerase chain reaction (RT-qPCR) assays of enzyme activity and spray drying survival rate were used to verify the results. The findings contribute to knowledge of LAB spray drying protectants.

## 2. Materials and Methods

### 2.1. Strains and Culture Conditions

*Lactobacillus delbrueckii* subsp. *bulgaricus* sp1.1 (*L. bulgaricus* sp1.1, CGMCC: 16586) was stored in De Man, Rogosa and Sharpe (MRS, Solarbio, Beijing, China) broth containing 20% (*v*/*v*) glycerol (Solarbio, Beijing, China) at −80 °C and 2% (*v*/*v*) aliquots that were subcultured in MRS broth at 37 °C for 12 h in the Laboratory of Probiotics and Fermented Food, Harbin Institute of Technology, Harbin, China [32]. After two 12 h growth periods, fresh medium at 2% (*v*/*v*) was added. The MRS broth was supplemented with 10 mM CaCl_2_ (MRSC, Solarbio, Beijing, China) or 10 mM MgCl_2_ (MRSM, Solarbio, Beijing, China), where indicated, with salts added immediately prior to cell culture.

### 2.2. Preparation of Feed Solutions for Spray Drying

Suspensions of *L. bulgaricus* sp1.1 were centrifuged at 6000× *g* at 4 °C for 15 min before the pellet was washed twice with 0.85% (*w*/*v*) saline solution and suspended in 30% (*w*/*w*) reconstituted skimmed milk (RSM, Nestle, Beijing, China). Micron carbonyl iron powder at 3% (*w*/*w*) (CIP, Zhong Yan, Beijing, China) and 30% RSM were added with continuous mixing before storage at 4 °C for up to 10 h.

### 2.3. Sampling during Spray Drying

Feed suspensions were stirred and spray-dried in a co-current laboratory spray dryer (SD-1000, Eyela, Tokyo, Japan). Volumetric airflow was 0.48–0.51 m^3^/min and atomizing pressure was 130 kPa and the feed rate was 400–600 mL/h [9]. The inlet temperature was set to 120 °C and the outlet temperature was stabilized at 60 °C by changing the feed rate.

Sampling was performed as described previously [12]. Briefly, samples were collected with a pre-cooled sampling device containing magnets and refrigerants, sodium polyacrylate and carboxymethylcellulose sodium (Solarbio, Beijing, China), to cool the sample. A sample of 151 ± 27 mg was taken for measurement of water content after drying. The sampling device was pre-loaded with 2 mL sterile water to rehydrate bacteria for the measurement of cell survival. Four sampling points were selected at 10 cm intervals (10, 20, 30 and 40 cm) inside the drying tower (tower height: 0.5 m). The 0 cm samples were controls (feed liquid suspension), and the 50 cm samples were powder in the cyclone chamber.

### 2.4. Cell Counts and Water Content

Samples were dried in a crucible at 105 °C for 6 h and water content calculated on a dry basis without CIP (g/g) [12] with the equation:

(1) $$water\;content\%=\frac{w_{0}-w_{i}}{w_{0}}*100\%$$

where *w*_0_ is the sample mass before drying and *w_i_* is the sample mass after drying.

Rehydrated samples were serially diluted in 0.01 M phosphate-buffered saline (PBS, Solarbio, Beijing, China) for culture on MRS agar at 37 °C for 48 h, followed by cell counting. Live bacteria were counted as the number of colonies per gram of dry matter (CFU/g) [9].

(2) $$survival\;rate\%=\frac{c_{i}}{c_{0}}*100\%$$

where *c*_0_ is the number of colonies per gram of dry matter (CFU/g) before drying and *c_i_* is the number of colonies per gram of dry matter (CFU/g) after drying.

### 2.5. Protein Extraction

The extraction of protein was performed as described previously [33]. Cells were washed with 50 mM Tris buffer (Solarbio, Beijing, China) and lysis buffer (8 M urea and 1% SDS with protease inhibitor, Solarbio, Beijing, China) was added before sonication in an ice bath until clear and centrifugation at 10,000× *g* for 30 min. The supernatant was loaded onto a 5% concentrated gel and 12.5% separation gel for SDS-PAGE [33]. The gel was stained with Coomassie Blue, and the Bradford method was used to quantify the protein concentration [34].

### 2.6. Protein Digestion and TMT Labelling

Protein digestion was achieved using the filter-aided sample preparation method [35]. In brief, a 100 μg protein sample was added to 90 μL lysis buffer, containing a final concentration of 100 mM triethylammonium bicarbonate buffer (TEAB) and 10 mM Tris (2-carboxyethyl) phosphine, and incubated at 37 °C for 1 h. Iodoacetamide to a final concentration of 40 mM was added before further incubation for 40 min. Pre-cooled acetone (acetone: sample volume ratio = 6:1) was added for precipitation at −20 °C for 4 h. The precipitate was dissolved in 50 mM TEAB. Trypsin (enzyme: protein = 1:50) was added for hydrolysis at 37 °C for 12 h.

Peptides were labeled with Tandem Mass Tag (TMT) six-plex reagents (Thermo Fisher, Waltham, MA, USA) by the addition of the TMT reagent to the polypeptide with incubation at room temperature for 2 h. Hydroxylamine was added with incubation for a further 15 min.

### 2.7. HPLC Fractionation and LC-MS/MS Analysis

An ACQUITY UPLC BEH C18 Column (Waters BEH C18 2.1 × 150 mm, 1.7 µm) and an Easy-1200 (Thermo, MA, USA) were used to perform liquid chromatographic reversed-phase separation of the pre-fractionated fractions. In short, peptides were combined into 20 fractions and a gradient of 2% to 80% acetonitrile was used to separate them. The chromatographic gradient was generated from solution A (2% acetonitrile and 0.1% formic acid) and solution B (80% acetonitrile and 0.1% formic acid) with a flow rate of 300 nL/min. Elution proceeded as follows: 0–1 min: 0–5% B; 1–63 min: 5–23% B; 63–88 min: 23–48% B; 88–89 min: 48–100% B; 89–95 min: 100% B. The mass spectrometer used for the LC-MS/MS was Q Exactive HF-X (Thermo, MA, USA).

Proteins with at least one unique peptide were identified with a false discovery rate (FDR) of <1.0%. The Mann–Whitney Test was used for statistical analysis of differentially expressed proteins (DEP) with a significant difference ratio of *p* < 0.05 and a fold change >1.2 or <0.83 was recorded.

### 2.8. Bioinformatic Analysis

GO analysis was performed using the InterPro scan-5 program on the non-redundant protein database with enrichment analysis using the GO enrichment pipeline. The PPI network was constructed using the STRING database with Cytoscape software (3.9.1).

### 2.9. RT-PCR Analysis

Gene-specific primer sets were designed with reference to NCBI Genbank and used for the amplification of target genes. RNA was extracted with the total RNA purification kit (GeneMark, Beijing, China) and cDNAs were synthesized using an All-in-One™ first-strand cDNA synthesis kit (GeneCopoeia, Rockville, Md, USA). RT-PCR was carried out using 2× All-in-One™ qPCR Mix (GeneCopoeia, Rockville, Md, USA) with 16S rRNA, acetyl-coenzyme A carboxylase carboxyl transferase subunit beta (*accD*), enoyl-[acyl-carrier-protein] reductase [NADH] (*FabI*), fructose-bisphosphate aldolase class-II (*FBA*), aspartate-semialdehyde dehydrogenase (*asd*), peptide ABC transporter permease (*oppBII*), magnesium-translocating P-type ATPase related genes (Ldb0341) and calcium-transporting ATPase related genes (Ldb0456). Details are given in Supplementary Table S1. 16S rRNA was the housekeeping gene [36]. Cycle threshold (CT) results were determined using the 2^−ΔΔCT^ method.

### 2.10. Detection of ATPase Activity

Cells were washed and lysed in ice-cold water for 10 min using an ultrasonic cell crusher (VCX105PB, SONICS, Newtown, USA) with a 20% intermittent pulse of 3 s. Ca^++^/Mg^++^ ATPase activities were measured using a Ca^++^/Mg^++^-ATP Kit (Solarbio, Beijing, China). One unit of enzyme activity is defined as the decomposition of ATP to produce 1μmol inorganic phosphate per 10^6^ cells per hour [37].

### 2.11. Intracellular Concentrations of Ca^++^ and Mg^++^

Cells were washed and digested with 4 mL 67% HNO_3_ for 4 h at 85 °C [38] with the evaporation of excess acid, followed by dilution with 5 mL 5% HNO_3_. Intracellular Ca^++^ and Mg^++^ concentrations were measured with an inductively coupled plasma optical emission spectrometer (ICP-OES, Agilent 730, Waldbronn, Germany).

### 2.12. Statistical Analysis

All data were the result of at least 3 independent experiments and were analyzed with SPSS 22 software (IBM, Armonk, NY, USA). The Mann–Whitney Test was used for the statistical analysis of DEPs. Tukey’s HSD test was used to test differences between groups, and the threshold for statistical significance was *p* < 0.05. Charts were drawn using Origin Pro 2018.

## 3. Results

### 3.1. Determination of the Sampling Point

**Figure 1:**
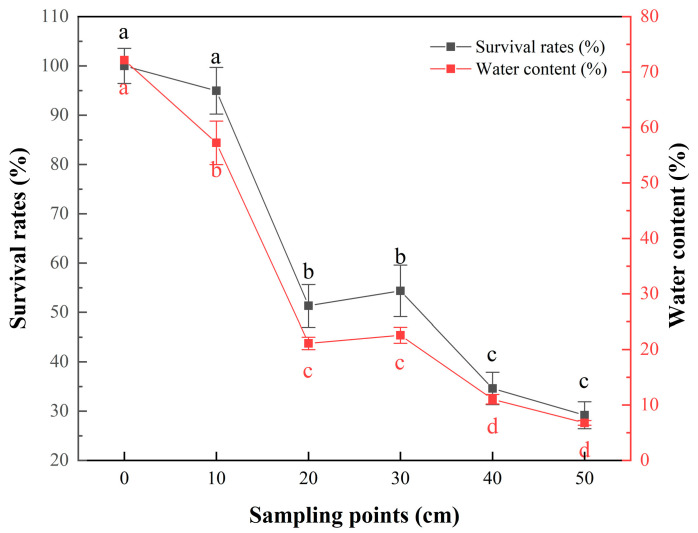
The relationship between cell water content and survival rate during spray drying. *p* < 0.05: differences between groups indicated by a, b, c and d.

The survival rate decreased with decreasing moisture content (Figure 1). The initial moisture content was 72.14% (0 cm) and decreased to 66.34%, 21.10%, 22.56%, 11.04% and 6.77% at 10, 20, 30, 40 and 50 cm, respectively. The corresponding survival rates were 94.96%, 51.31%, 54.36%, 34.60% and 29.17%. The samples at 20 cm were the first point where survival significantly decreased, and there was no significant change (*p* > 0.05) in the survival rate between 20 cm and 30 cm. There was also no significant change (*p* > 0.05) in the survival rate between 40 cm and 50 cm. The sample at 50 cm was the final product of spray-dried bacteria, which was more convenient for sampling, so the samples at 20 cm and 50 cm were selected as the sampling points for proteomic analysis.

### 3.2. Protein Identification and GO Analysis of DEPs

**Figure 2:**
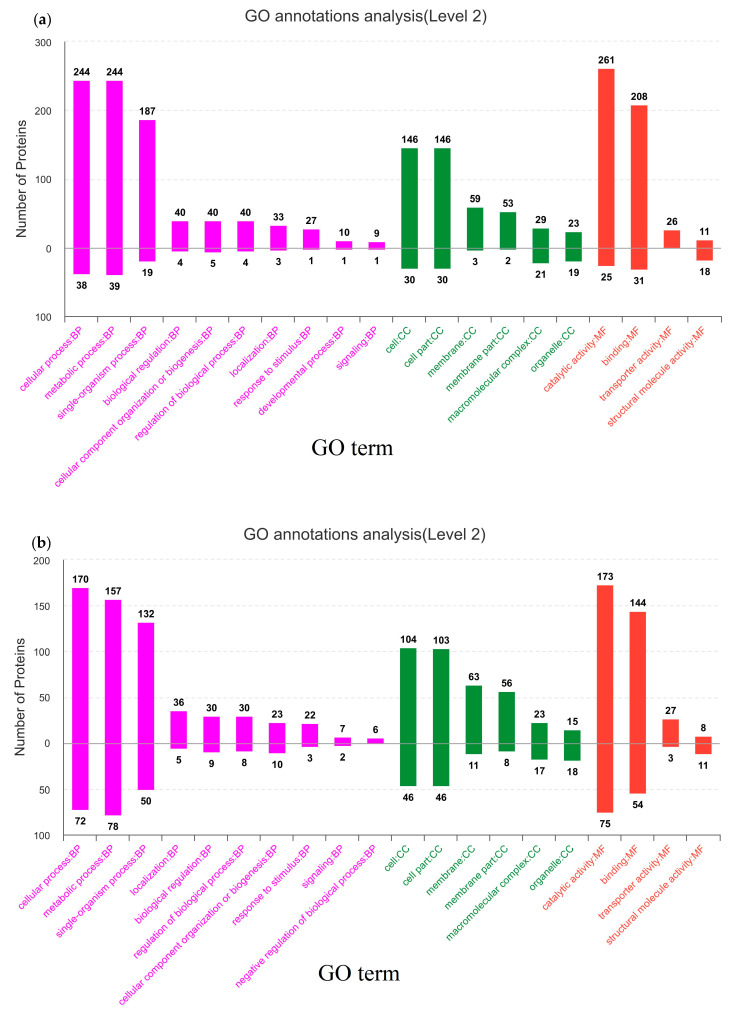

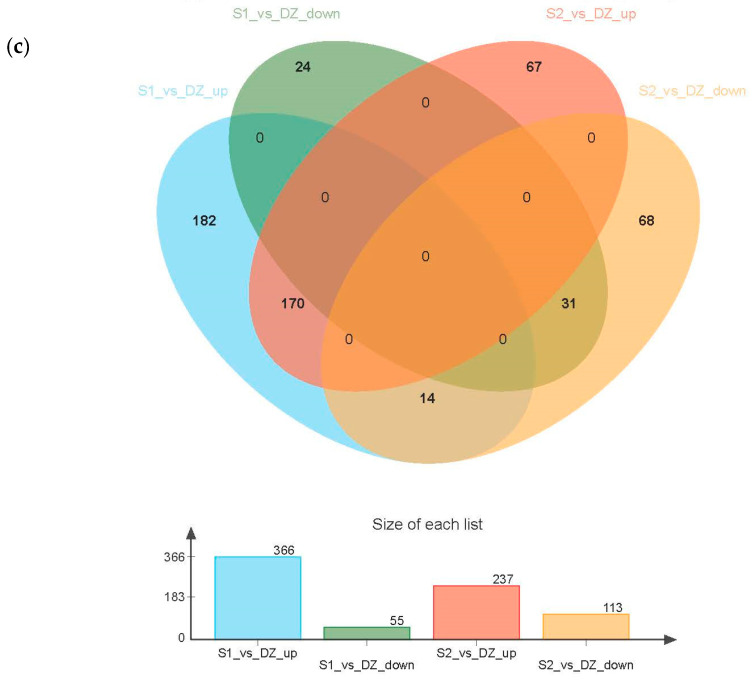
GO analysis of DEPs. (**a**) GO analysis of DEPs at the 20 cm sampling point. (**b**) GO analysis of DEPs at the 50 cm sampling point. (**c**) Venn diagram comparing up- and down-regulated proteins. (**a**,**b**): column represents GO classification with annotation of secondary GO classifications of proteins shown by the direction of the bar. Height represents the number of proteins in the secondary classification. Abscissa represents the GO second-level classification terms. The abscissa represented the GO second-level classification terms. (**c**): S1_vs_DZ_up: proteins up-regulated at the 20 cm sampling point; S1_vs_DZ_down: proteins down-regulated at the 20 cm sampling point; S2_vs_DZ_up: proteins up-regulated at the 50 cm sampling point; S2_vs_DZ_down: proteins down-regulated at the 50 cm sampling point.

*L. bulgaricus* sp1.1 DEPs were analyzed at 0, 20 and 50 cm during spray drying (Figure 2a,b) with 0 cm samples being the control. In total, 908 bacterial proteins were identified. A total of 421 DEPs were found in the 20 cm samples, of which 366 were up-regulated and 55 were down-regulated. A total of 350 DEPs were found in the 50 cm sample, of which 237 were up-regulated and 113 were down-regulated (Figure 2c).

A GO analysis classification of DEPs was performed to associate proteins with molecular function (MF), biological process (BP) and cell composition (CC). In the 20 cm and 50 cm samples, changes in metabolic, cellular and single-organism processes, cell parts, catalytic activity and binding were shown by the occurrence of DEPs.

### 3.3. Functional Enrichment Analysis and PPI Network Construction

**Figure 3:**
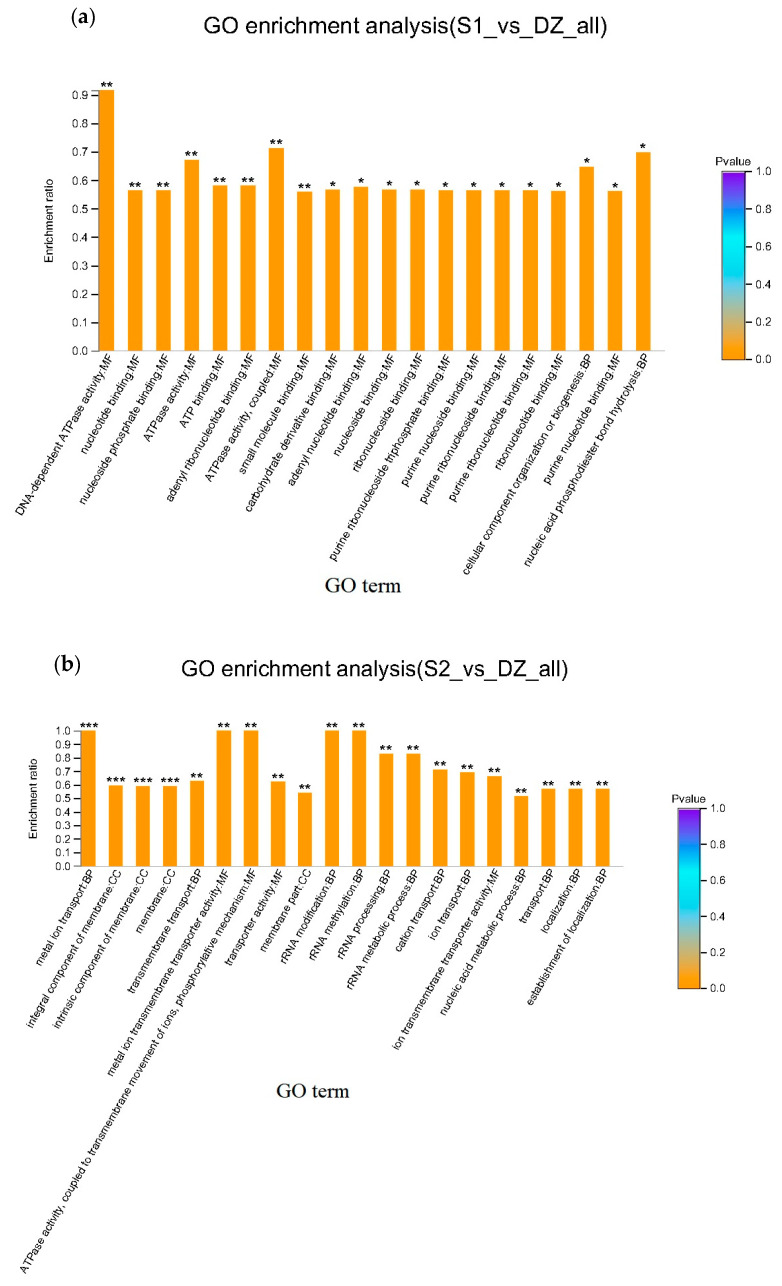
GO functional enrichment analysis of DEPs. (**a**) GO functional enrichment analysis of DEPs at the 20 cm sampling point. (**b**) GO functional enrichment analysis of DEPs at the 50 cm sampling point. S1_vs_DZ_all: proteins at the 20 cm sampling point; S2_vs_DZ_all: proteins at the 50 cm sampling point. Abscissa: GO term; ordinate: enrichment rate. The ratio of the number of proteins enriched in the GO term: background number annotated by the GO term is shown. The color gradient of the column indicates the significance of enrichment; *p* < 0.05: *; *p* < 0.01: **; *p* < 0.001: ***.

The GO analysis indicated enrichment of the pathways, including DNA-dependent ATPase activity, nucleotide binding, nucleoside phosphate binding, ATP binding, adenyl ribonucleotide binding, ATPase activity and coupled and small molecule binding in the 20 cm samples (*p* < 0.01, Figure 3a). Thus, most changes were related to DNA replication and repair. Proteins related to metal ion transport and integral and intrinsic membrane components were enriched in the 50 cm samples (*p* < 0.001, Figure 3b). Thus, most changes were related to membranes and transport. DEPs related to the transport of potassium, calcium and magnesium ions were particularly enriched.

**Figure 4:**
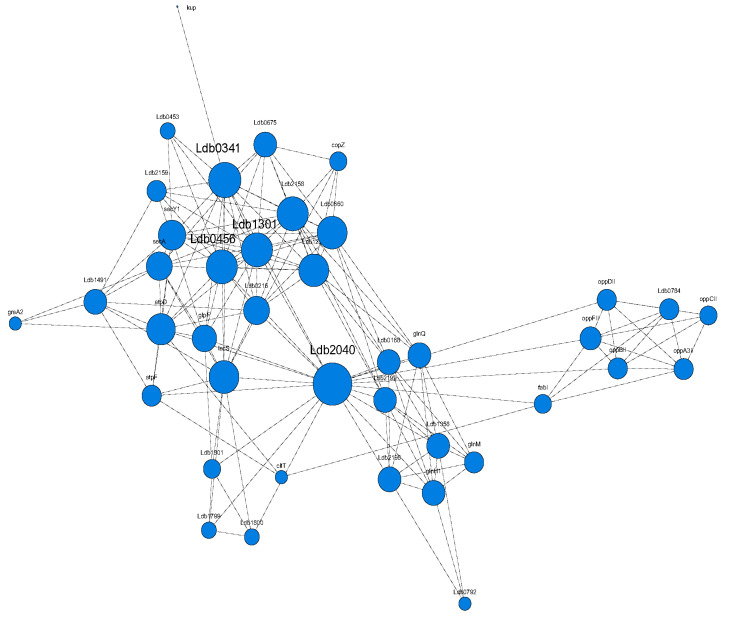
PPI network of DEPs. Proteins are represented by nodes and interactions between proteins by edges. The larger the node, the greater the importance of the node gene in the network.

A PPI network of interactions among differently expressed transport proteins was constructed. Ldb2040 (PTS fructose transporter subunit IIC), Ldb0341 (magnesium-translocating P-type ATPase), Ldb1301 (metal ABC transporter ATPase) and Ldb0456 (calcium-transporting ATPase) showed a high degree of connectivity to other proteins (Figure 4).

### 3.4. Proteomic Data Validation Using RT-qPCR

**Figure 5:**
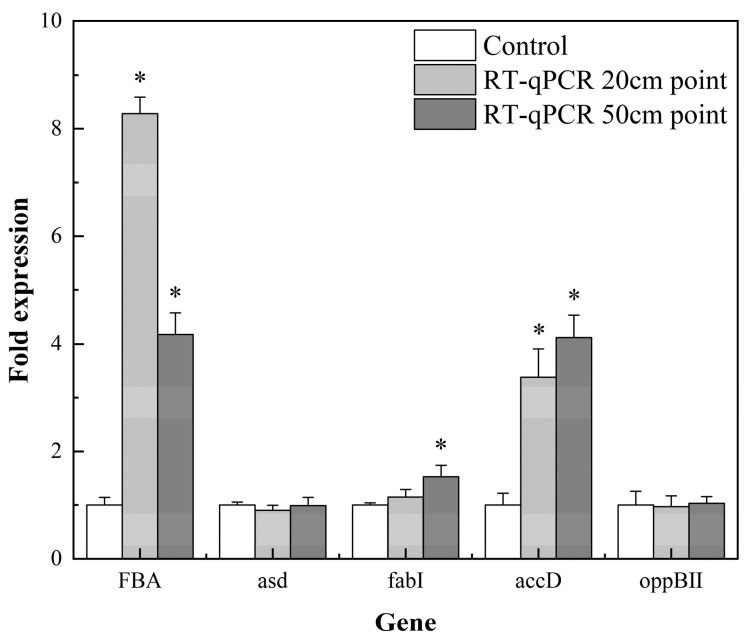
Fold expression of different genes. RT-qPCR results. The limits of up- and down-regulation are 1.2 and 0.83, respectively. *p* < 0.05: *.

The GO pathway genes including *accD*, *FabI*, *FBA*, *asd* and *oppBII* were assessed with RT qPCR to verify the TMT results (Figure 5). The genes *accD*, *FabI*, *FBA* and *oppBII* were expressed in concordance with the protein abundance while *asd* expression was inconsistent with the protein abundance in the 20 cm samples. Expression levels of *asd*, *FabI* and *accD* agreed with the proteomics results, but *FBA* and *oppBII* did not in the 50 cm samples.

### 3.5. Ca^++^/Mg^++^ ATPase Activity and Expression of Related Genes during Spray Drying

**Figure 6:**
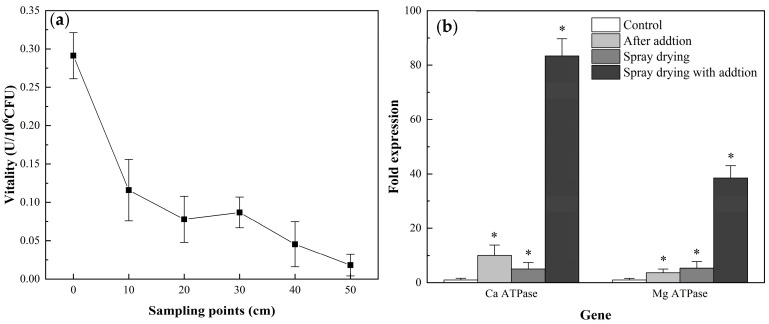

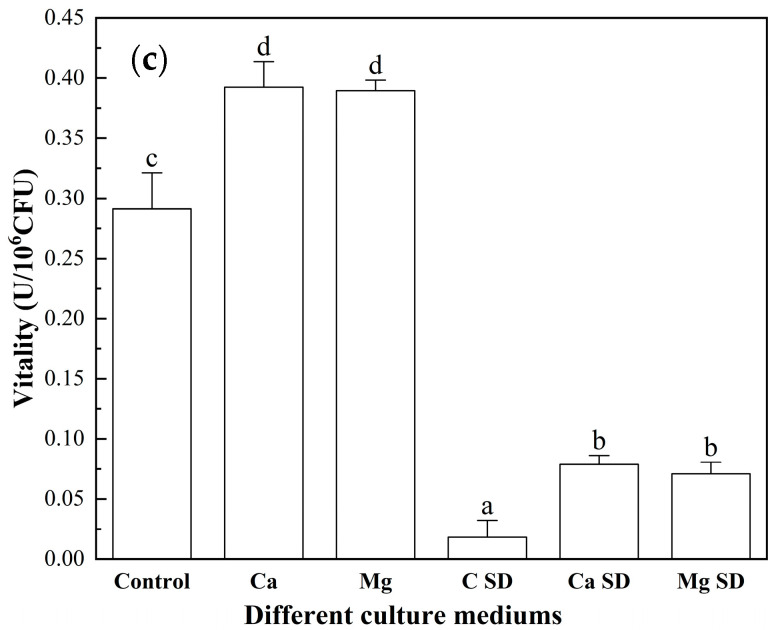
Ca^++^/Mg^++^ ATPase activity and expression of related genes during spray drying. (**a**) Changes in Ca^++^/Mg^++^ ATPase activity during spray drying. (**b**) Expression of Ca++/Mg++ ATPase-related genes after supplementation with CaCl_2_ and MgCl_2_. *p* < 0.05: *. (**c**) Ca^++^/Mg^++^ ATPase activity after spray drying. *p* < 0.05: differences between groups indicated by a, b, c and d.

Ca^++^/Mg^++^ ATPase activity was gradually lost during spray drying, being greatly reduced at the 0–20 cm sampling points (Figure 6a) and almost completely lost at the 50 cm sampling point. The loss of Ca^++^/Mg^++^ ATPase activity may account for a substantial proportion of LAB spray drying damage.

When *L. bulgaricus* sp1.1 was cultured in MRSC or MRSM for 12 h to regulate the expression of Ca^++^/Mg^++^ ATPase, it was found that the addition of CaCl_2_ or MgCl_2_ increased the expression of ATPase related genes (Figure 6b). The expression of calcium-transporting ATPase related genes increased 10.07-fold, and the expression of magnesium-translocating P-type ATPase related genes increased 3.65-fold. Ca^++^/Mg^++^ ATPase activity increased from 0.29 U (control) to 0.392 U (MRSC) or 0.389 U (MRSM; Figure 6c). After spray drying (50 cm sampling point), the activities of Ca^++^/Mg^++^ ATPase were significantly higher (*p* < 0.05) than controls (0 cm sampling point). Expressions of calcium-transporting ATPase related genes were increased 83.33-fold and that of magnesium-translocating P-type ATPase related genes were increased 38.5-fold.

### 3.6. Intracellular Concentrations of Ca^++^ and Mg^++^ and Survival Rate

**Figure 7:**
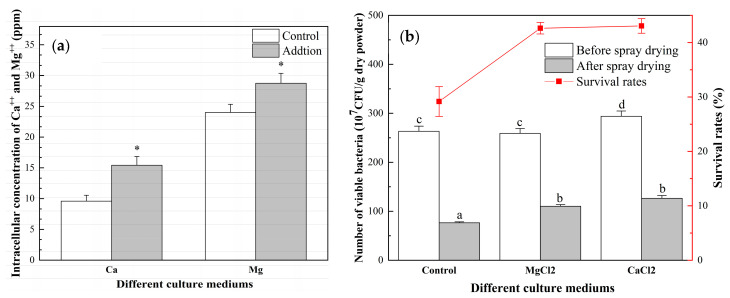
Intracellular Ca^++^ and Mg^++^ concentrations and survival rate. (**a**) Intracellular Ca^++^ and Mg^++^ concentrations. *p* < 0.05: *. (**b**) Spray-drying survival rate after supplementation with CaCl_2_ and MgCl_2_. *p* < 0.05: differences between groups indicated by a, b, c and d.

The culture of *L. bulgaricus* sp1.1 in MRSC increased the intracellular calcium ion concentration to 15.42 ppm compared with 9.60 ppm in the controls (Figure 7a). Similarly, the culture of *L. bulgaricus* sp1.1 in MRSM increased the intracellular magnesium ion concentration to 28.76 ppm compared with 24.01 ppm for the controls.

Following the elevation of intracellular calcium and magnesium ion concentrations, the survival rates after spray drying were 43.06% and 42.64%, respectively. These values were 13.89% and 13.47% higher than the survival rates of the controls (Figure 7b).

## 4. Discussion

Compared with single-drop drying, laboratory spray dryers had shorter residence time [3]. The new sampling method was used to analyze the changes in protein expression of *L. bulgaricus* sp1.1 during spray drying. The bacterial survival rate decreased with the reduction in water content, and the water content due to spray drying of 21.10% water was a critical threshold. Below 25.28% water, there is little bacterial survival in the spray drying process [12]. Previous work has shown a drastic decrease in the survival of *Lactobacillus helveticus* below a water content of about 0.3–0.5 g/g [39], consistent with the findings of the present study. At the point of exit from the atomizer, the droplet has sufficient moisture content and kinetic energy to move along the original linear direction without being affected by the lateral airflow [40]. As the droplet descends, it experiences increased air resistance and its mass is reduced. When the force of the lateral motion of the air cannot be overcome, the droplet will be pulled upwards. It will enter the recirculation vortex area of the inner wall and circulate along the inner wall of the drying tower [41]. The low outlet temperature will increase the moving distance of the particles in the main circulation area and prolong the drying time to meet the need for low moisture content of the dried powder (Goula and Adamopoulos, 2004). These movements led to a slight increase in water content and survival rate at the 30 cm sampling point compared with the 20 cm sampling point.

When the survival rate dropped from 100% to 57.24%, proteins related to DNA replication and repair were differentially expressed, although DNA was not damaged during the drying process. Changes in DNA-related proteins may have been brought about due to the stress response [42]. On reduction from 57.24% to 29.17%, membrane-associated proteins were differentially expressed, consistent with previous results. Reduction of the water content to <0.31 g/g proved lethal to most bacteria due to the disruption of membranes [9]. There was little correlation between survival and the GO analysis. However, increased expression of Ca^++^/Mg^++^ ATPase was found in both the 20 cm and 50 cm samples. Spray drying caused peroxidation of the membrane lipid bilayers and alterations in the physical state of lipids on the cellular membrane [43,44]. Previous studies [9] have shown that DNA is not damaged during spray drying, and the damage is mainly concentrated on the membrane and membrane transport. Therefore, the 50 cm samples were selected for follow-up studies.

Expression of *asd* mRNA at the 20 cm sampling point was inconsistent with the abundance of *asd*-related proteins, and the expression of *FBA* and *oppBII* mRNA at the 50 cm sampling point was also inconsistent with the results of the proteomics analysis. Discrepancies in the correlation between protein and mRNA expression may be due to altered rates of transcription, post-translational modification and protein degradation [45,46].

In the last few years, there has been an increased focus on the ability of Ca^++^ to provide thermal protection for LAB [16]. When *Lactococcus lactis* subsp. *cremoris* (*L. cremoris*) were cultured with CaCl_2_, the cells showed an increase in intracellular calcium ion concentration [47]. An elevation in intracellular Ca^++^ activated heat shock proteins, potentially increasing the thermal tolerance of cells [16,47]. Enhanced thermotolerance was shown by *L. cremoris* and *Lactobacillus acidophilus* (*L. acidophilus*) grown in M17 medium containing 10 mM CaCl_2_ to promote intracellular Ca^++^ accumulation, as shown using a murexide assay [47]. The survival rate of spray-dried *L. cremoris* increased from 49.9% to 64.9% and that of *L. acidophilus* from 35.5% to 43.3%. Adding Ca^++^ to milk can induce a large amount of milk protein aggregation after heat treatment, thus protecting LAB cells during spray drying [48]. Our results also showed that the addition of calcium and magnesium ions increased the survival of spray-dried *L. bulgaricus* sp 1.1.

Ca^++^/Mg^++^ ATPase was gradually inactivated during spray drying. Ca^++^ ATPase is a cell membrane calcium pump necessary to maintain intracellular Ca^++^ concentration for cell function [49]. Antioxidant xanthan-oligosaccharide is known to inhibit *Staphylococcus aureus* growth by increasing cell membrane permeability and reducing Ca^++^/Mg^++^ ATPase activity [50]. Moreover, high extracellular Ca^++^ concentration has been shown to up-regulate proteins involved in Ca^++^ efflux [51]. Metal ion homeostasis, especially that of divalent cations, is known to regulate the survival of various bacterial species under heat stress [52] and addition of Mg^++^ or Ca^++^ has been shown to increase LGG survival of heat stress [21,27]. However, our research found that the addition of Ca^++^ and Mg^++^ promoted the expression of Ca^++^/Mg^++^ ATPase related genes. Adding Ca^++^ or Mg^++^ enhanced Ca^++^/Mg^++^ ATPase activity, thus increasing the survival of spray-dried *L. bulgaricus* sp 1.1.

The mRNA levels of Ca^++^/Mg^++^ ATPase were lower after the addition of Ca^++^ or Mg^++^, although enzyme activities were raised ~4.98 and ~5.49 fold, respectively. Correlations between protein and mRNA levels were poor, probably as a result of altering rates of transcription, post-translational modifications and protein degradation [45,46]. Previous studies have revealed similar results. Oral administration of Al decreased Na^+^/K^+^ ATPase activity in Kunming mouse kidneys, while mRNA expression increased [53]. Furthermore, the recovery of *L. bulgaricus* from spray drying damage was shown to take place without protein synthesis [54], indicating either that the synthesis of new protein was not required or that bacterial damage impaired the process of protein synthesis but not that of transcription [8,55].

## 5. Conclusions

When the water content reached 21.10%, the ability of bacteria to endure spray drying decreased drastically. Ca^++^/Mg^++^ ATPase was deactivated during the spray drying process, which may have contributed to the decreased survival rate of bacteria. The addition of Ca^++^ and Mg^++^ during bacterial culture increased the expression of genes related to calcium-transporting and magnesium-translocating P-type ATPases and enhanced Ca^++^/Mg^++^ ATPase activity. Intracellular Ca^++^ and Mg^++^ concentrations were also increased, as was the survival rate.

## Data Availability

The datasets generated during and/or analyzed during the current study are available from the corresponding author on reasonable request. Proteome data (https://www.iprox.cn/page/home.html, accessed on 6 March 2020, Username: sunjialei, Password: Sjl1994!).

## Supplementary Materials

The following supporting information can be downloaded at: https://www.mdpi.com/article/10.3390/foods12040787/s1, Table S1: Primers of *L. bulgaricus* sp1.1
