# Supplementary material for: The Effect of Decreased Ca++/Mg++ ATPase Activity on Lactobacillus delbrueckii subsp. bulgaricus sp1.1 Survival during Spray Drying
Source: Foods. 2023 Feb 13;12(4):787. doi: 10.3390/foods12040787 (PMC9955740; doi:10.3390/foods12040787)
Supplement: Supplementary file 1 [file foods-12-00787-s001.zip › foods-2157492-supplementary.pdf]

Supplementary Table S1 Primers of *L. bulgaricus* sp1.1

| Primers | Sequences (5′–3′) | Bases | Length (bp) |
|---|---|---|---|
| *accD* F | CGAGGAAAGCGAGGAGTG | 18 | 400 |
| *accD* R | GGTAGTCGGGTCGGTCAAA | 19 | |
| *FabI* F | GGTCCTTGACGGTTGATG | 18 | 108 |
| *FabI* R | CGTAGATGATGTCGCCAGT | 19 | |
| *FBA* F | GGTGCCTACAACACTAACA | 19 | 169 |
| *FBA* R | AGATGTCCATAGCGTCCA | 18 | |
| *16S rRNA* F | TACCAAGGCAATGATGCGTA | 20 | 123 |
| *16S rRNA* R | CATCAGACTTGCGTCCATTG | 20 | |
| *Ldb0341* F | CAAGGAATCAGCGGACAT | 18 | 176 |
| *Ldb0341* R | GCAAGAATGGCAGGAAGA | 18 | |
| *Ldb0456* F | CCGACGACTGACAATGTT | 18 | 320 |
| *Ldb0456* R | GCCTTCACGATTCTAACCT | 19 | |

The primer sequences used for RT-PCR. Primers of 16S rRNA, acetyl-coenzyme A carboxylase carboxyl transferase subunit beta (accD), enoyl-[acyl-carrier-protein] reductase [NADH] (FabI), fructose-bisphosphate aldolase class-II (FBA), aspartate-semialdehyde dehydrogenase (asd), peptide ABC transporter permease (oppB Ⅱ), magnesium-translocating P-type ATPase related genes (Ldb0341) and calcium-transporting ATPase related genes (Ldb0456).
